# Integrating Single Domain Antibodies into Field-Deployable Rapid Assays

**DOI:** 10.3390/antib11040064

**Published:** 2022-10-17

**Authors:** George P. Anderson, Lisa C. Shriver-Lake, Jinny L. Liu, Ellen R. Goldman

**Affiliations:** US Naval Research Laboratory, Center for Biomolecular Science and Engineering, 4555 Overlook Ave SW, Washington, DC 20375, USA

**Keywords:** vertical flow assay, single domain antibody, gold nanoparticle, staphylococcal enterotoxin B, ricin, SARS-CoV-2 nucleocapsid

## Abstract

Single domain antibodies (sdAb) are the recombinant variable heavy domains derived from camelid heavy-chain antibodies. While they have binding affinities equivalent to conventional antibodies, sdAb are only one-tenth the size and possess numerous advantages such as excellent thermal stability with the ability to refold following denaturation, and inexpensive production in *Escherichia coli* or yeast. However, their small size does have drawbacks, one being that they can lose activity upon attachment or adsorption to surfaces, or may fail to adsorb efficiently, as they are highly soluble. This can make the transition from using conventional antibodies to sdAb nontrivial for assay development. Specifically, it is often necessary to re-optimize the protocols and tailor the recombinant sdAb through protein engineering to function efficiently in handheld assays, which currently are utilized for point of care testing and field applications. This work focuses on optimizing the integration of sdAb into rapid vertical flow assays. To achieve this goal, we engineered sdAb-based constructs and developed general protocols for the attachment of the sdAb to both gold nanoparticles and a support membrane. We achieved a limit of detection of 0.11 µg/mL for toxins staphylococcal enterotoxin B and ricin, both potential biothreat agents. Additionally, we demonstrated the ability to detect the nucleocapsid protein of SARS-CoV-2, a common target of antigen tests for COVID-19.

## 1. Introduction

The objective of this work was to elucidate methods whereby single domain antibodies (sdAb) could be integrated into field-deployable rapid hand-held diagnostic and detection assays. A lateral flow assay is a simple-to-use diagnostic device for determining the presence or absence of target analytes, such as pathogens or contaminants in water supplies. The most commonly known type of lateral flow assay is the pregnancy test. Rapid, low cost, portable, and easy to use, lateral flow assays are used in biomedicine, agriculture, food and environmental sciences [1]; they are currently the basis for most rapid tests for COVID-19 [2]. Lateral flow assays require that the recognition elements be adsorbed to both a membrane, typically made of nitrocellulose, and to colloidal gold nanoparticles. An alternative format for rapid assays that has been demonstrated for the multiplexed and sensitive detection of biothreat agents, is the vertical flow assay (VFA) which also requires recognition elements adsorbed to both a membrane and gold nanoparticles [3]. Antibodies, which are routinely employed as the recognition elements for these tests, must form stable interactions with the surfaces while retaining their binding activity.

SdAb, also known as nanobodies or VHH, are the recombinantly produced binding domains derived from the unique heavy-chain-only antibodies found in camelids [4,5]. Like conventional antibodies, sdAb provide sensitive and specific binding to target analytes, but are only 1/10 the size. Advantages of sdAb include the ability to refold and bind antigen after denaturation, to bind hidden epitopes, to be readily produced in bacterial or yeast systems, and to be engineered for specific applications [6,7]. SdAb have been developed towards an ever-growing list of high value targets, and they are being utilized for a number of therapeutic and detection applications [8,9,10].

The methods and materials for lateral and vertical flow assays have traditionally been optimized for conventional antibodies. Because of their inherent stability, and economical production, small and rugged sdAb provide an alternative to conventional antibodies, however, little is known on how well sdAb can substitute for conventional antibodies in these assay formats. It is almost certain that for a sdAb to function optimally will require that the protocol or the sdAb itself be tailored to enhance its ability to stably adsorb and retain high activity [11]. Protein engineering can be used to create fusions of sdAb with either peptides or proteins to promote stable, directional, and active immobilization [12,13,14]. The elucidation of the optimal sdAb fusion constructs to enable their facile use for rapid hand-held lateral flow assays would help facilitate their widespread adoption.

Herein, we developed protocols to facilitate the integration of sdAb into vertical flow assays (VFA). First, we focused on optimizing an assay for the toxin, staphylococcal enterotoxin B (SEB), a potential biothreat agent. We had previously developed a pair of highly stable sdAb for SEB detection. Both were stable up to at least 70 °C and they functioned efficiently as a sandwich pair in other assay formats [12]. These two sdAb were optimized for use in a vertical flow assay that, similar to lateral flow assays consists of one sdAb immobilized on a membrane and another onto the surface of a gold nanoparticle. The optimal sdAb fusion protein required was different for each of the surfaces. Once we validated the optimal fusion constructs, we looked to demonstrate the universal applicability of our approach by preparing fusion construct with sdAb specific for other targets. While the sdAb format selected for coating the membrane was transferable to new assays, as observed previously preparation of the gold nanoparticles can be more problematic [11]. To address this issue, an alternative approach, which promised to provide a more universal solution for coating sdAb onto gold nanoparticles, was pursued.

## 2. Materials and Methods

### 2.1. Reagents

Unless otherwise specified, chemical reagents were from Sigma Aldrich (St. Louis, MO, USA), Thermo Fisher Scientific (Waltham, MA, USA), or VWR International (Radnor, PA, USA). Restriction endonucleases and ligation reagents were from New England Biolabs (Ipswich, MA, USA). Eurofins Genomics (Louisville, KY, USA) performed oligonucleotide synthesis and DNA sequencing. The SEB-binding monoclonal antibody 2F2 was the kind gift of Dr. Thomas O’Brien, Tetracore, Rockville, MD. SEB was from Toxin Technology (Sarasota FL, USA), ricin was from Vector Laboratories (Newark, CA, USA), and recombinant nucleocapsid from SARS-CoV-2 (N) was from ACRO Biosystems (Newark, DE, USA). The following reagents were obtained through BEI Resources, NIAID, NIH: Staphylococcal Enterotoxin B Toxoid, Recombinant from *Escherichia coli*, NR-10049, and is referred to as SEBv.

All sdAb and variants were in pET22b expression vectors; typically, sdAb were cloned as *NcoI-NotI* fragments. The anti-SEB sdAb ACVE and A3H2 and anti-ricin sdAb D12f and F6H2Y had been described previously [15,16,17,18], including genetically linked versions of ACVE, a bivalent version of ACVE linked with a SpyTag (ST), and a series of ACVE variants with synthetic zipper (synzip) tails [12,19,20,21]. The anti-SARS-CoV-2 nucleocapsid (N) sdAb (E2, C2, and B6) and the genetically linked C2B6 construct have also been recently described [22], along with variants of E2, C2, and B6 that had a C-terminal hop tail (GAGGSGGAPASNRCSQGSCWN) [22,23].

The sequences of the C-terminal tails used in this work are provided in the supplemental information (Supplemental Table S1), and the sequences of the sdAb are provided in Supplemental Table S2.

### 2.2. SdAb Fusions and Nomenclature

A number of new sdAb variants were constructed. Versions of sdAb ACVE were prepared in which two gold-binding peptides (gbps; gbp1: MHGKTQATSGTIQS [24]; gpb2: LKAHLPPSRLPS [25]) were synthesized as *NotI*-*XhoI* fragments and cloned into pET22b for the expression of fusions with sdAb, which was cloned as *NcoI-NotI*. Variants of sdAb ACVE and A3H2 with the hop tail (GAGGSGGAPASNRCSQGSCWN) [23] were prepared as described [26]. A bivalent form of A3H2 was prepared following a strategy described previously in which the first sdAb is flanked by *NcoI-NotI* restriction sites and the second sdAb flanked by *BamHI-XhoI* restriction sites with a “GGGGSGGGGSGGGGS” linker between them [22]. Versions of the sdAb genetically linked to rhizavidin (RZ; a dimeric biotin binding protein) were prepared as previously described [13]; RZ fusions were prepared for all the SEB and ricin binding sdAb as well as for E2, one of the SARS-CoV-2 N binding sdAb. Each new construct was verified by DNA sequencing.

Fusion proteins are named with the sdAb name, a hyphen, and the name of the fusion. Genetic fusions of the sdAb ACVE with the hop tail are called ACVE-hop. A bivalent construct where two ACVE are genetically linked is ACVE-ACVE, and a fusion with rhizavidin would be termed ACVE-RZ. In all cases a glycine-serine containing linker bridges the parts of the fusion protein.

### 2.3. Protein Production

The pET22b-based sdAb expression plasmids were transformed into Tuner (DE3) for protein production. Freshly transformed colonies were used to start overnight cultures in 50 mL terrific broth (TB) containing ampicillin (100 µg/mL) at 25 °C. The next day, the overnight cultures were poured into 450 mL of TB with ampicillin and grown for 2 h at 25 °C prior to induction with isopropyl-D-1 thiogalactoside (IPTG, 0.5 mM) and a further 2 h growth. Purification was carried out through an osmotic shock protocol followed by immobilized metal affinity chromatography (IMAC) and fast protein liquid chromatography (FPLC) as described previously [27]. The concentration of each sdAb preparation was determined by UV absorption and sdAb preparations were kept refrigerated for immediate use or aliquoted and frozen at −80 °C for long-term storage.

### 2.4. Preparation of Vertical Flow Membranes

The Miriad™ test cartridges obtained from Cytodiagnostics (Burlington, ON, Canada) were typically coated with one or two capture sdAb (1.5 µL at 1 mg/mL) for the target being analyzed along with a spot of Goat anti-VHH polyclonal antibody (1 µL at 0.6 mg/mL) (Jackson ImmunoResearch Labortories Inc., West Grove, PA, USA). Some cartridges also had NeutrAvidin (1 µL at 1 mg/mL) as an additional control. Thus, each cartridge had between two to four capture spots. After the binding elements were spotted onto the membrane it was allowed to dry at RT for 1 h and then blocked using 1 mg/mL bovine serum albumin (BSA) in PBS, also for at least 1 h.

### 2.5. Adsorption of sdAb Constructs to Gold Nanoparticles

The initial protocol to coat the gold nanoparticles (AuNPs) was optimized to work for sdAb constructs that included a hop tail. While this protocol works with those same sdAb in the absence of the hop tail, better coating was observed with this modification. A solution of sdAb-hop (final concentration—0.5 mg/mL) was diluted in water with 1 mM TCEP (tris(2-carboxyethyl)phosphine) a reducing agent, and 5 mM sodium borate pH 9.0. This mixture was allowed to incubate for five minutes before a 1/10th volume was added to the AuNPs. The preferred AuNP for these experiments were the 20 nM stabilized AuNP from Cytodiagnotics. The sdAb was incubated with the AuNPs for 15 min, and then centrifuged at 20 K·rpm for 5 min at 8 °C. The sdAb-AuNPs were resuspended in distilled water to their original volume and then another 1/10th volume of TCEP-reduced sdAb was added and incubated for an additional 15 min before centrifuging as before. The sdAb-AuNPs were then washed twice by resuspending in the original volume using 10 mM Borate pH 9.0, 0.025% Tween-20, and 1 mg/mL BSA (wash buffer). The sdAb-coated AuNPs were resuspended in PBS plus 0.05% Tween-20 (PBST) with 1 mg/mL BSA after the second wash and stored at 4 °C until used.

### 2.6. Conjugation of sdAb-RZ to Gold Nanoparticles Coated with Biotinylated Proteins

In this approach, the AuNPs were first coated with a biotinylated protein and then an sdAb–rhizavidin (RZ) fusion was used to orient the sdAb onto surfaces [13]. The biotinylated proteins evaluated for this approach were sheep IgG, rabbit IgG, goat IgG, BSA, casein, and ovalbumin (OVA). Protein was biotinylated with a 10-fold molar excess biotin-LC-NHS ester for 30 min, then free biotin was removed using a Zeba spin column. A similar protocol to that described in Section 2.5 was adopted for the adsorption of the biotinylated (Bt)-proteins. Bt-protein (1 mg/mL) was reacted with TCEP (1 mM) for five minutes and then added to 20 nM standard AuNPs from Cytodiagnotics at a concentration of 25 µg/mL. The Bt-protein was incubated with the AuNPs for 15 min, and then centrifuged at 20 K rpm for 5 min at 8 °C. The AuNPs were resuspended using distilled water to their original volume and then another aliquot of Bt-protein (25 µg/mL final) was added and incubated for 15 min before centrifuging as before. The AuNPs were resuspended in water and the desired sdAb-RZ fusion added at 50 µg/mL for 30 min. Next a 1/10 volume of 10 mg/mL BSA, 10 mM sodium borate (pH 9.0) was added prior to centrifuging as before. The sdAb-coated AuNPs were then washed twice by resuspending in the starting volume using the wash buffer described above. Finally, the sdAb-coated AuNPs were resuspended in PBST and 1 mg/mL BSA and stored at 4 °C until used.

### 2.7. Vertical Flow Assay (VFA)

To perform the vertical flow assay, the Miriad™ test cartridge that had been coated with the desired proteins and blocked with BSA was first washed with 0.1 mL of universal buffer (Cytodiagnostics). Then 0.1 mL of the target antigen in PBST was applied. After 1 min, the cartridge was washed with 0.1 mL of universal buffer. Then, 0.1 mL of sdAb-coated AuNPs were applied to the cartridge and allowed to sit for 1 min prior to washing twice with 0.1 mL of universal buffer. The cartridge was visually inspected and photographed for the presence of both the control and assay spot.

## 3. Results and Discussion

### 3.1. Optimizing a VFA Using Anti-SEB sdAb

Similar to lateral flow, the VFA format consists of a capture reagent immobilized on a membrane and a reporter reagent immobilized on a gold particle, however the fluids are applied to the top surface of the membrane and pass vertically through rather than flowing parallel across [28]. VFAs have been developed for the detection of potential biothreat agents such as *Burkholderia pseudomallei* [3]. VFAs are potentially easier to be multiplexed than lateral flow assays, and offer a simpler system for testing protocols for coupling of sdAb to membranes and gold. The major limitation of the vertical flow format is the reduced contact time of the sample and reagents with the surface, hence only a limited portion of these materials will contact the capture spot, while a significantly larger proportion makes contact in the lateral flow format. Thus, it is expected that substantially improved detection limits would be realized upon converting the assays described here to the lateral flow format.

We had previously engineered two anti-SEB sdAb to have improved stability resulting in clones ACVE and A3H2 [15,16,19]. These sdAb were demonstrated to work well as a pair in sandwich assays for SEB. Also, we had previously constructed a number of ACVE variants including bivalent and trivalent constructs, as well as fusions with synthetic zipper (synzip) and SpyTag (ST) tails [12,19]. In addition, these sdAb recognize both SEB toxin, and SEBv, a non-toxic mutant. Therefore, we selected this pair of sdAb to evaluate for incorporation into the VFA format.

Our first objective was to determine the optimal sdAb construct for immobilization onto the membrane. The Miriad™ test cartridges from Cytodiagnostics facilitated the side-by-side evaluation of four different ACVE fusion constructs at a time. In this test we evaluated: ACVE, ACVE-gbp1, ACVE-gbp2, ACVE-ACVE, ACVE-ACVE-ACVE, ACVE-hop, ACVE-ACVE-ST, ACVE-E34, ACVE-synzip 5, ACVE-synzip 6, ACVE-synzip 17, and ACVE-synzip 18. Each cartridge was spotted with 1.5 µL of four different ACVE constructs (1 mg/mL each), challenged with 100 µL of SEB at 10 µg/mL. The relative amount SEB captured was determined using AuNPs coated with a conventional anti-SEB monoclonal antibody. From these initial tests, the three most promising, ACVE-ACVE, ACVE-ACVE-ST, and ACVE-synthetic zipper 17 were tested again versus the baseline ACVE (Supplemental Figure S1). While all three of these constructs functioned well, we selected the dimer ACVE-ACVE as the capture molecule of choice for two reasons: (1) being a larger molecule with two binding sites it seemed reasonable to believe it would adsorb better than the monomer with an increased probability that at least one of the two binding sites would remain active, (2) being that preparation of dimer constructs is a relatively common solution to enhancing binding via avidity it may have more utility than the other peptide fusions evaluated.

Next, our focus turned towards determining a facile method for the adsorption of sdAb to the AuNP surface. While AuNPs are available with specialized surfaces that allow attachment via chemical crosslinking, our objective was to develop a low-cost method compatible with standard citrate stabilized AuNP reagents. Prior efforts had succeeded in immobilizing sdAb to AuNPs by optimizing the solution pH during attachment [11]. We hoped to avoid this process individualization by identifying a fusion construct for which a single attachment protocol could be utilized. First fusions of ACVE with two different gold binding peptides and with a hop tail were tested for their ability to coat gold particles. The hop tail is a peptide tag that contains a short linker and two cysteine residues plus an amino acid sequence for substrate recognition by *E. coli* disulfide isomerase [23]. This tag was originally designed to facilitate the oriented attachment of antibody binding fragments to surfaces, such as gold particles, without compromising the protein production of the antibody binding fragments. Both ACVE-gbp versions caused aggregation of the gold particles, so the hop tail version was pursued for optimization of the gold-sdAb reagent. For initial tests of the reagent, SEBv was spotted on the membrane, and we observed the ability of the ACVE-hop coated AuNPs to bind. Goossens et al. (2017) [11] had evaluated sdAb constructs that had a single C-terminal Cys residue, but found no benefit in their hands. However, we found the ACVE-hop to work better than other constructs tested (Supplemental Figure S2).

**Figure 1:**
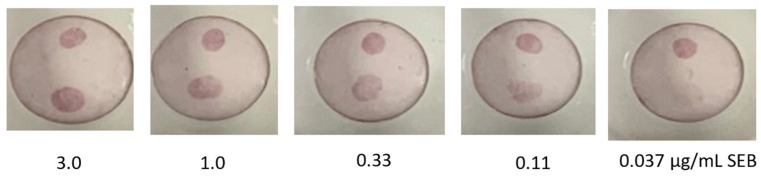
Vertical flow assay for the detection of SEB using A3H2-hop coated AuNPs. Each Miriad test cartridge was spotted with ACVE-ACVE near the bottom and goat anti-VHH near the top, as a control. After each cartridge was challenged with 100 µL of SEB at the indicated concentration, 100 µL of A3H2-hop AuNP was applied to generate the colored spot.

Next, we combined the ACVE-ACVE capture spots with the A3H2-coated AuNPs for SEB detection. Preliminary experiments compared the preparation of AuNPs using A3H2 and A3H2-hop. Since the version with the hop-tail appeared to coat more effectively, the protocol was further optimized for the A3H2-hop construct. In addition, it was thought that better attachment might be achievable to the AuNPs if the disulfide bond of the hop-tail was reduced prior to adsorption onto the AuNPs. While this did appear to be true, based on prior work it is difficult to ascribe the exact mechanism of action as TCEP is known to impact the manner biomolecules interact with AuNPs [29]. Using an adsorption protocol for coating of stabilized 20 nM AuNPs from Cytodiagnostics using A3H2-hop, a dose curve for the detection of SEB was evaluated using ACVE-ACVE as the capture molecule on the Miriad™ test cartridges (Figure 1). A limit of detection of 0.11 µg/mL was achieved, which for this rapid format where interaction time is limited was considered successful. In the future, potentially lower limits of detection could be realized in a VFA system utilizing membranes of a different pore size that increases contact time [3].

### 3.2. Examining sdAb-Rhizavidin Fusion as a Universal Method to Coat Gold Nanoparticles

While the protocol using a sdAb-hop fusion construct initially looked promising, when attempts were made to coat AuNP with other sdAb-hop fusions (i.e., E2-hop, C2-hop, and B6-hop) it was quickly apparent that this approach was far from universal, as half of the sdAb-hop constructs tested caused the AuNP to aggregate. To investigate this result in more detail, we examined the ability of pI mutants of our chikungunya binding sdAb (CC3-hop) to coat AuNPs effectively. These 10 mutants had pI’s that varied from 6.1 to 9.2 [26]. Of these 10, only two appeared to coat AuNP stably, the ones with a pI of 8.75 and 9. Thus, it became clear that use of sdAb-hop fusions would not allow a facile solution to preparation of sdAb-coated AuNPs. While pI is a factor it was not the only factor as the other hop tail fusions did not obey this simple model, making it difficult to know *a priori* if a sdAb-hop tail fusion would coat the AuNPs successfully.

In a bid to attain a universal protocol, we took an alternative approach wherein the AuNPs would first be coated with a biotinylated protein. We first utilized sheep IgG, assuming any biotinylated protein that coats AuNPs effectively would suffice. Once a biotin layer was adsorbed to the surface, the sdAb could then be attached by use of sdAb-RZ fusion protein. Previously, we had shown that sdAb-RZ fusions produce efficiently in *E. coli* and provide an effective means to create a bifunctional molecule, having both antigen and biotin binding motifs [13]. Another beneficial feature for this application is that RZ forms a homodimer, thus most of the sdAb-RZ constructs should attach to the biotinylated protein on the AuNPs bivalently, thereby creating a highly stable interaction. In addition, RZ is stable to 100 °C; thus, a thermally resistant interaction as well [30].

**Figure 2:**
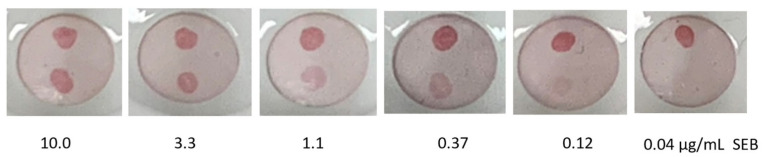
Vertical flow assay for the detection of SEB using A3H2-RZ coated AuNPs. Each Miriad test cartridge was spotted with ACVE-ACVE near the bottom and goat anti-VHH near the top, as a control. After each cartridge was challenged with 100 µL of SEB at the indicated concentration, 100 µL of A3H2-RZ-Bt-sheep-IgG-AuNP was applied to generate the colored spot.

The initial assay for SEB was evaluated using A3H2-RZ to coat AuNPs that had first been coated with Bt-sheep-IgG. This approach yielded detection down to 0.12 µg/mL, equal to that observed using the A3H2-hop coated AuNPs, indicating that preparation of sdAb-coated AuNPs using this method was at least as effective (Figure 2).

**Figure 3:**
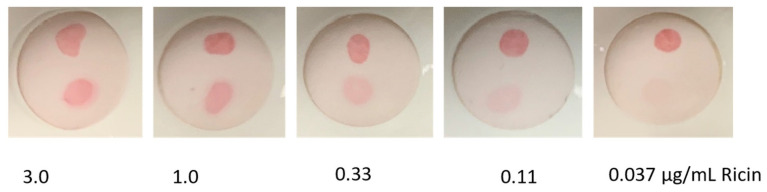
Vertical flow assay for the detection of Ricin using D12f-RZ coated AuNPs. Each Miriad test cartridge was spotted with F6H2Y anti-ricin sdAb near the bottom and goat anti-VHH near the top, as a control. After each cartridge was challenged with 100 µL of ricin at the indicated concentration, 100 µL of D12f-RZ-Bt-sheep-IgG-AuNP was applied to generate the colored spot.

Our next goal was to examine if the use of sdAb-RZ constructs truly represented a universal approach for the preparation of sdAb-coated AuNPs. To test this hypothesis, we turned to another assay and sdAb reagents we had developed previously: those for the detection of ricin. Two ricin A chain binding sdAb that work well as a sandwich pair were selected for use. F6H2Y was adsorbed to the Miriad test cartridges while the D12f-RZ was coated onto the Bt-sheep-IgG-AuNPs. For this assay, we successfully obtained a limit of detection for ricin of at least 0.11 µg/mL, satisfactory for most rapid detection needs, since relative to bacterial and viral threats, a large quantity of ricin would be needed to constitute a biothreat (Figure 3).

### 3.3. VFA for Nucleocapsid (N) of SARS-CoV-2

As a final demonstration of the capacity to integrate sdAb into VFAs using methods developed here, we examined the ability to detect SARS-CoV-2 N. Based on our MagPlex sandwich assay results for the detection of N using sdAb we had developed previously [22], we tested their ability to be utilized in the VFA format using the E2 sdAb to coat the AuNP as a RZ fusion and C2B6 a heterobivalent sdAb as the capture sdAb on the membrane. When using the AuNPs coated with the Bt-sheep IgG, we observed that the E2-RZ-Bt-sheep-IgG-AuNP aggregated during storage. Thus, while our first two assays were successful using the Bt-sheep-IgG to coat the AuNPs, failure using with E2-RZ indicated that our goal of achieving a universal method was not realized.

**Figure 4:**
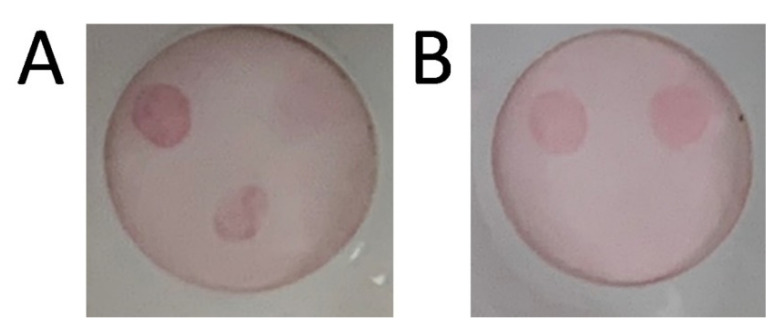
VFA for SARS-CoV-2 N detection using E2-RZ coated AuNPs. Both Miriad test cartridges were spotted with bivalent C2-B6 sdAb near the bottom, goat anti-VHH top left, and NeutrAvidin top right. After each cartridge was challenged with 100 µL of N at 3 µg/mL, 100 µL of E2-RZ-Bt-Ovalbumin-AuNP (panel (**A**)) or 100 µL of E2-RZ-Bt-caseine (panel (**B**)) was applied to generate the colored spot.

To understand and solve this conundrum, we first considered that the net charge on the sdAb-RZ construct was to blame. To test this hypothesis, we compared the ability of several different sdAb-RZ constructs to prepare stable AuNPs in conjunction with a Bt-sheep-IgG base layer. It was observed that approximately half of the constructs were stable, while the other half led to aggregated AuNPs with no correlation with net charge. Since the net charge of the sdAb-RZ fusion did not seem to be responsible for the failure, we turned our attention to the base lay and prepared a range of biotinylated proteins to test as the base layer with the E2-RZ construct that had failed with the Bt-sheep-IgG. The protein biotinylated for this test were rabbit-IgG, goat-IgG, BSA, casein, and ovalbumin (OVA). In conjunction with the E2-RZ, only the Bt-casein and Bt-OVA provided stable bioconjugated AuNPs. Both were used to evaluate their ability to function in the VFA. Figure 4 shows the results of that trial. The AuNP prepared with the casein base layer bound to both control spots indicating that both Bt-casein and the E2-RZ were on the surface, however no binding was observed on the C2B6 spot, indicating that the resultant E2-RZ was either not being bound in a suitable conformation or in sufficient amounts. This is contrasted with the results from the Bt-OVA-coated AuNPs, where the anti-VHH control spot is prominent, with a much weaker Neutravidin spot, suggesting a more complete coverage of the E2-RZ on the surface. More importantly, the C2B6 capture spot was found to be active, and the detection of SARS-CoV-2 N was successful. Lower concentrations were not evaluated for this assay since to reach the requisite limits of detection one would likely need to optimize the VFA configuration and support membrane or perform lateral flow assays. The other sdAb-RZ constructs that had failed to remain soluble with Bt-sheep-IgG coated AuNPs, were also found to be compatible with the Bt-OVA-coated AuNPs. Thus, Bt-OVA appears to provide the universal base layer coating on AuNP that we had desired. It is possible that the degree of biotinylation plays a role in how stable this surface is. More work will be needed to ascertain why the Bt-OVA was successful where the other candidate Bt-proteins failed, and perhaps a more exhaustive search would identify an even better Bt-protein to serve as the base layer, but for now the Bt-OVA provides the universal solution sought.

## 4. Conclusions

Currently, sdAb have been reported towards a wide range of toxin, bacterial, and viral targets, with the list growing. Many sdAb fusions have been described that improve the utility of sdAb, and much has been done to develop an understanding of how to engineer enhanced thermal stability into sdAb. Through this work, we have developed an understanding of the best constructs and methods for the immobilization of sdAb onto membranes and gold particles, both critical to achieve successful handheld assays with any sdAb. Fusions of sdAb with peptides designed to promote conjugation to gold, such as the hop tail, did not provide a universal method of producing sdAb-AuNP reagents. However, use of a genetic fusion between sdAb and rhizavidin appears to provide a more general way of immobilizing onto gold particles with adsorbed Bt-OVA.

We demonstrated the detection of two toxins, ricin and SEB, using a VFA and showed initial feasibility of integrating sdAb constructs into a VFA for detection of SARS-CoV-2. SdAb have the potential to provide a reliable source of biothreat detection reagents engineered for functionality in austere locations. The ability to incorporate sdAb into handheld assays may enhance the shelf-life of the assays and thereby decrease the logistical burden to field biodetection assays.

## Data Availability

The data presented in this study are contained within the article and supplementary material.

## Supplementary Materials

The following supporting information can be downloaded at: https://www.mdpi.com/article/10.3390/antib11040064/s1, Table S1. Tail names and amino acid sequences. Table S2. Sequences of sdAb used in the VFA. Figure S1. Evaluation of different ACVE capture formats. Figure S2. Evaluation of two ACVE-based constructs on AuNPs.
