# Supplementary material for: Integrating Single Domain Antibodies into Field-Deployable Rapid Assays
Source: Antibodies (Basel). 2022 Oct 17;11(4):64. doi: 10.3390/antib11040064 (PMC9624348; doi:10.3390/antib11040064)
Supplement: Supplementary file 1 [file antibodies-11-00064-s001.zip › antibodies-1926658-supplementary.pdf]

Supplementary material

Integrating Single Domain Antibodies into Field-Deployable Rapid Assays

George P. Anderson, Lisa C. Shriver-Lake, Jinny L. Liu, and Ellen R. Goldman*

US Naval Research Laboratory, Center for Biomolecular Science and Engineering, 4555 Overlook Ave SW, Washington, DC 20375, USA

Table S1. Tail names and amino acid sequences

Table S2. Sequences of sdAb used in the VFA

Figure S1. Evaluation of different ACVE capture formats

Figure S2. Evaluation of two ACVE-based constructs on AuNPs

Table S1. Tail names and amino acid sequences

Tail name	Tail sequence
Hop tail	GAGGSGGAPASNRCSQGSCWN
Spy tag (ST)	AHIVMVDAYKPTK
Gold binding peptide (gbp) 1	MHGKTQATSGTIQS
Gbp2	LKAHLPPSRLPS
Synthetic zipper (synzip) 5	NTVKELKNYIQELEERNAELKNLKEHLKFAKAELEFELAAHKFE
synzip 6	QKVAQLKNRVAYKLKENAKLENIVARLENDNANLEKDIANLEKDIANLERDVAR
synzip 17	NEKEELKSKKAELRNRIEQLKQKREQLKQKIANLRKEIEAYK
synzip 18	SIAATLENDLARLENENARLEKDIANLERDLAKLEREEAYF
E34	ITIRAAFLEKENTALRTEIAELEKEVGRSENIVSKYETRYGPL
Rhizavidin (RZ)	FDASNFKDFSSIASASSSWQNQSGSTMIIQVDSFGNVSGQYVNRAQGTGCQN SPYPLTGRVNGTFIAFSVGWNNSTENCNSATGWTGYAQVNGNNTEIVTSWNL AYEGGSGPAIEQGQDTFQYVPTTENKSLLKD

Table S2. Sequences of sdAb used in the VFA

SdAb name	SdAb sequence
ACVE	DVQLVESGGGLVQPGGSLRLTCAASGLIFGSYAMGWFRQAPGKAREFVAAISWSGGDTYADSVK GRFTISRDNAKNTVYLQMNSLEPEDTAVYSCAAVGSKYYISKDAKDYGYWGQGTQVTVSS
A3H2	EVQLVESGGGLVQAGDSLRLSCTASGRIFSRAVMGWFRQAPGKEREFVAAISAAPGTAYYAFYADS VRGRFIISADSAKNTVYLQMNSLKPEDTAVYYCAADLKMQVAAYMNQRSVDYWGQGTQVTVSS
D12f	EVQLQASGGGLVQDGGSLRLSCAVAGRPLSDYGVGWFRQASGKEREFVAVISGSGIVTDYANSVK GRFTISRDVAKNVVHLQMNSLKPEDTAVYYCAALTNPVYAASRNSNDYGYWGQGTQVTVSS
F6H2Y	QVQLVESGGGLVQAGGSLRLSCATSGGTFSDYGMGWFRQAPGKEREFVAAIRRNGNGGNGIEYA DSVKGRFTISRDNAKNTVYLQMNSLTPEDTAVYYCAASISGYAYNTIERYNYWGQGTQVTVSS
E2	EVQLQASGGGLVQAGGSLRLSCAASGRTDSTQHMAWFRQAPGKEREFVTAIQWRGGGTSYTDS VKGRFTISRDNAKNTVYLEMNSLKPEDTAVYYCATNTRWTYFSPTVPDRYDYWGQGTQVTVSS
C2	EVQLQASGGGLVRPGGSLRLSCAASGFTFSSYAMMWVRQAPGKGLEWVSAINGGGGSTSYADS VKGRFTISRDNAKNTLYLQMNSLKPEDTAVYYCAKYQAAVHQEKEDYWGQGTQVTVSS
B6	EVQLQASGGGLVQAGDSLRLSCVAVSGRTISTFAMGWFRQAPGKEREFVATINWSGSSARYADPV EGRFTISRDDAKNTVYLEMSSLKPGDSAVYYCASGRYLGGITSYSQGDFAPWGQGTQVTVSS

Figure S1.

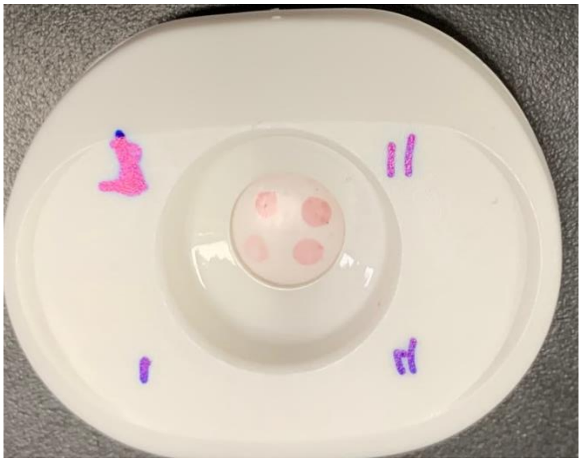

Evaluation of different ACVE capture formats. Upper left spot = ACVE; upper right spot = ACVE-ACVE; lower left spot = SCVE-ST; lower right spot = ACVE-synthetic zipper 17

Figure S2.

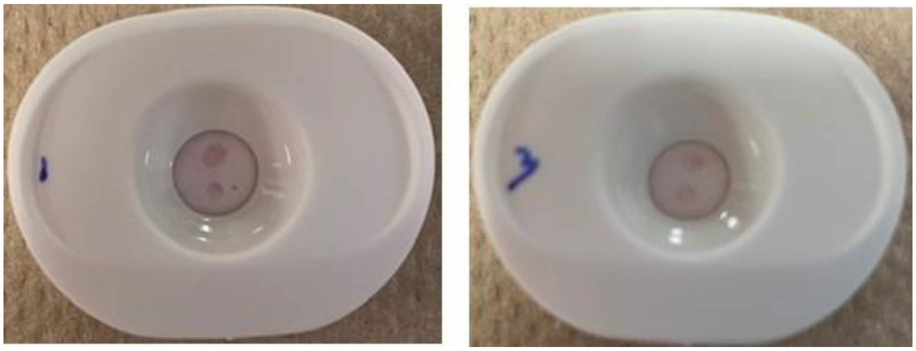

Evaluation of two ACVE-based constructs on AuNPs. Left is ACVE-hop and right is the trivalent construct ACVE-ACVE-ACVE. Miriad test cartridges were spotted with a monoclonal anti-SEB (mAb 2F2) on the top, and SEBv on the bottom. Next 100 µL of SEBv (10 µg/mL) was applied to the cartridge, followed by a wash and the AuNPs. Cartridges were washed again and photographed.
